# Stepwise multi-stage resection technique for surgical extraction of a partially erupted mandibular third molar with inferior alveolar nerve entrapment: a case report

**DOI:** 10.3389/froh.2024.1509998

**Published:** 2024-12-02

**Authors:** Mohammed A. Alsaegh, Elyazia Fayyad, Farah Jalal, Hana Mulhem, Manar Qasho, Nour Mohannad, Wissam Moughrabel

**Affiliations:** ^1^ Department of Oral and Craniofacial Health Sciences, College of Dental Medicine, University of Sharjah, Sharjah, United Arab Emirates; ^2^ Research Institute for Medical and Health Sciences, University of Sharjah, Sharjah, United Arab Emirates

**Keywords:** mandibular third molar, inferior alveolar nerve, surgical extraction, CBCT, entrapment, case report

## Abstract

The removal of lower third molars is one of the most common surgical procedures in routine dental practice. However, perforation of the mandibular third molars by the inferior alveolar nerve (IAN) is a rare occurrence. These cases are considered to carry a heightened risk of IAN injury due to the nerve being entrapped within the tooth. This case report details the experience of a 43-year-old female who visited the clinic for surgical removal of her lower third molar. She reported a six-month history of pain in area of tooth number 38, along with persistent hypersensitivity radiating through the lower lip and chin on the left side of her face, accompanied by abnormal sensations and numbness occurring alongside the pain. The case includes entrapment of IAN within the root of the partially erupted tooth, causing neurosensory disturbances. The associated lower third molar was extracted using a stepwise multi-stage resection technique in order to preserve the entrapped nerve. The patient's pain improved after surgery. This report contributes to current clinical knowledge and practices for extracting teeth with IAN entrapment. It addresses a gap in the limited and outdated literature on such cases.

## 1 Introduction

Compared to the ophthalmic and maxillary nerves, the mandibular division of the trigeminal nerve is more susceptible to damage during oral surgery (1). Within the mandibular division, the inferior alveolar nerve (IAN) serves as a sensory nerve, providing sensation to the mandibular bone, mandibular teeth, gingiva, buccal mucosa anterior to the mental foramen, and the soft tissues of the lower lip and chin (2). Oral surgeons are aware of potential two-plans radiographic signs indicating the IAN proximity to the adjacent teeth. These radiographic indicators include root darkening, diversion of the inferior alveolar canal, and obstruction of the mandibular canal, all of which suggest potential entrapment of the nerve by the third molar's roots during eruption (3). This proximity increases the risk of nerve injury and sensory deficits in the lower jaw and lip during surgical removal. To minimize this risk, further radiological evaluation is essential and can help optimize treatment outcomes (4). Advanced imaging techniques, such as cone beam computed tomography (CBCT), can clarify the anatomical relationship between the nerve and the surgical area, reducing uncertainty (4). Along with a comprehensive assessment, precise surgical technique is crucial when managing mandibular third molars that are in close proximity to the IAN.

Entrapment of the IAN within the roots of mandibular third molars is a rare occurrence, with the incidence of perforation estimated at around 1 in 800 impactions (5). However, clinicians who frequently perform mandibular third molar surgery often report encountering only 1–2 cases over their careers. This suggests that the true incidence may be lower than estimated or that such cases are frequently unrecognized (6). IAN entrapment can result in neuropathic pain (7). Several surgical techniques have been proposed for the removal of lower molars perforated by the IAN (7–11). The present case was managed at the University Dental Hospital of Sharjah. The report aims to present a rare case of IAN becoming entrapped within the root of a mandibular third molar, resulting in neuropathy. It details the patient's clinical presentation, symptoms, radiographical analysis, surgical intervention, and follow-up care.

## 2 Case description

**Figure 1:**
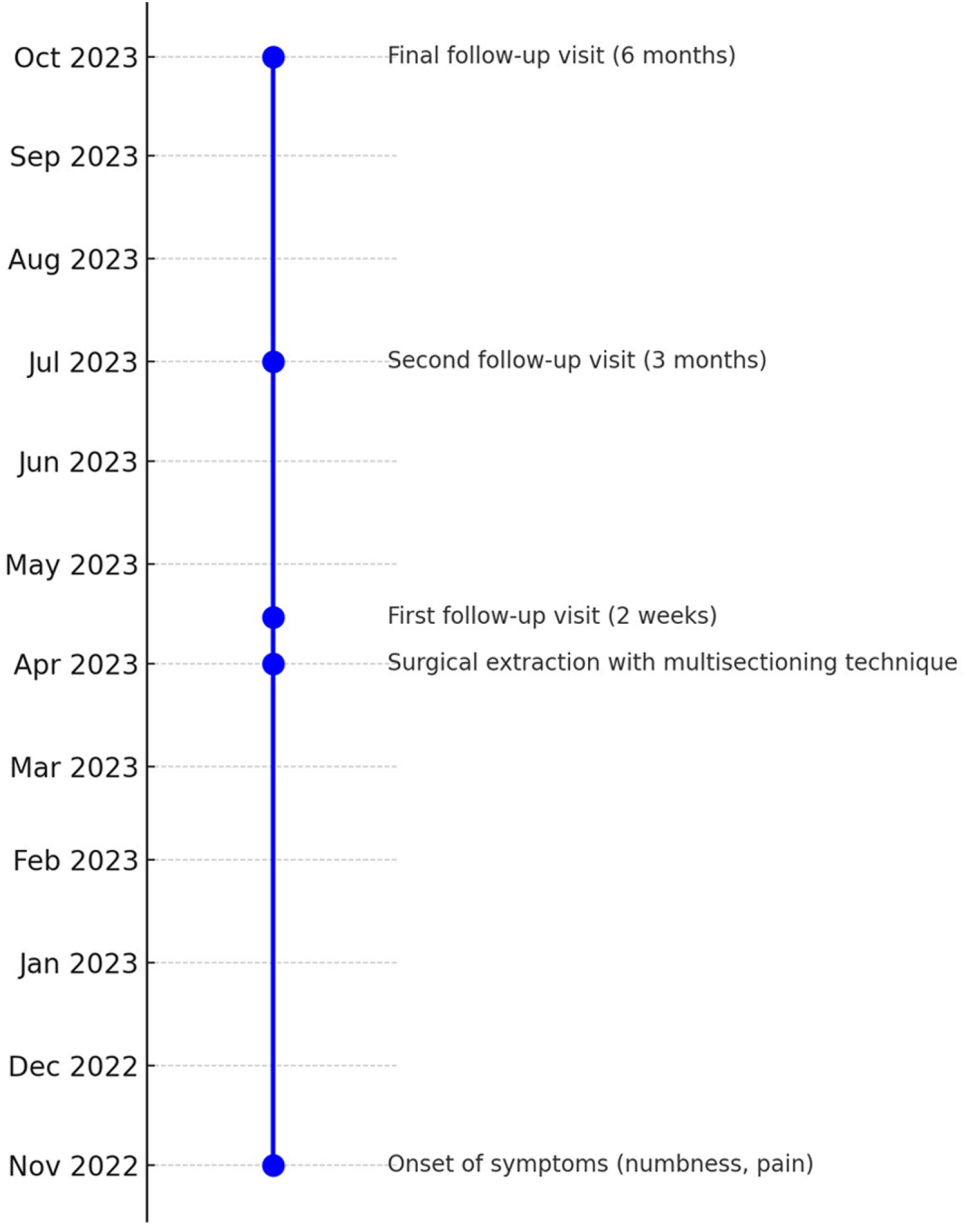
Timeline of the present case report.

**Figure 2:**
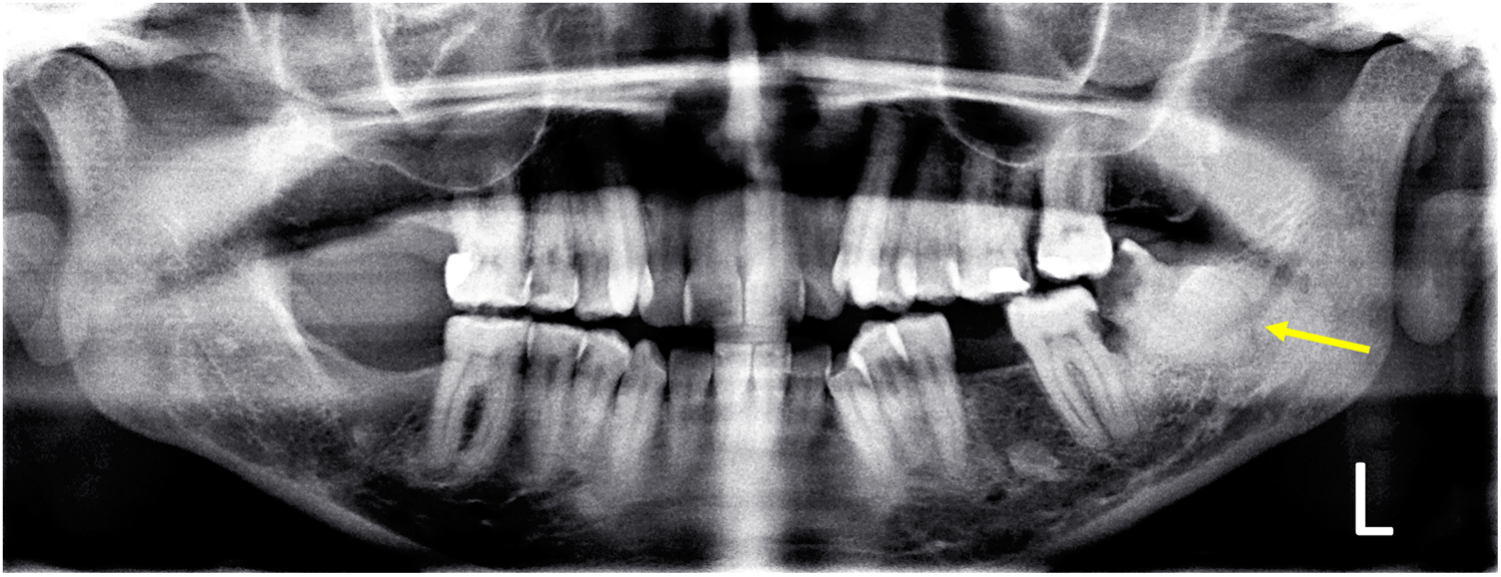
Preoperative orthopantomogram illustrates the relationship between the inferior alveolar bundle and the root of the mandibular left third molar. The relationship is predicted by darkening of the root and narrowing and upward deviation of the mandibular canal.

**Figure 3:**
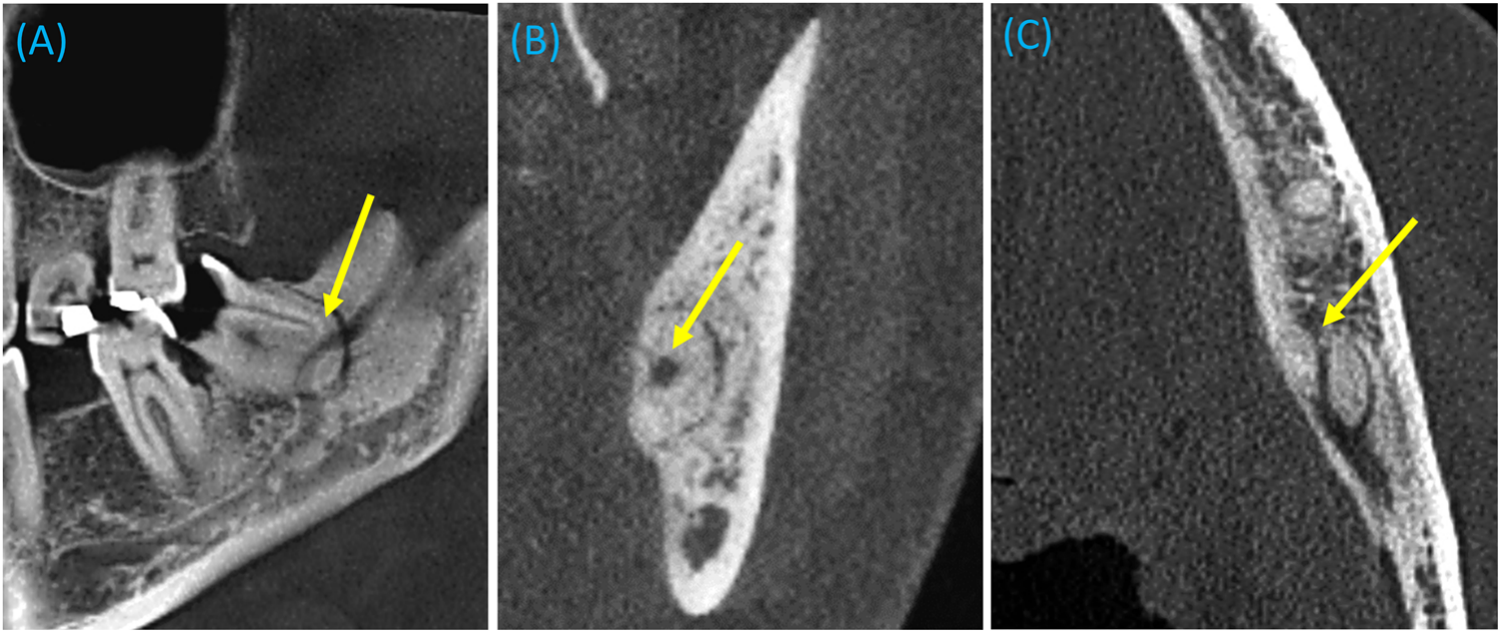
Preoperative cone beam computed tomography (1 mm section) reveals entrapment of the inferior alveolar canal within the fused root of the mandibular third molar. The arrows indicate the inferior alveolar canal in **(A)** sagittal, **(B)** coronal, and **(C)** axial sections.

**Figure 4:**
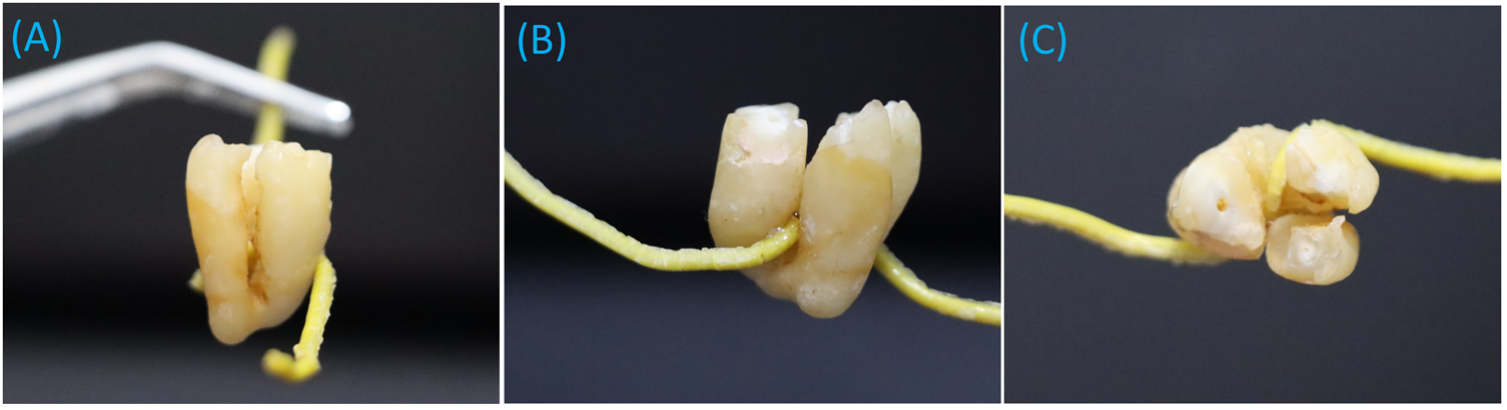
The extracted pieces are reconstructed using adhesive and a simulated wire to replicate the pathway of the inferior alveolar nerve, which is entrapped within the fused root of the tooth. Shown in **(A)** mesial view, **(B)** lingual view, and **(C)** occlusal view.

The timeline of the current case report is illustrated in Figure 1. A 43-year-old female presented to the University Dental Hospital of Sharjah with a history of pain in the area of tooth number 38, accompanied by persistent hypersensitivity radiating through the lower lip and chin on the left side of her face, with abnormal sensations and numbness alongside the pain. The pain occasionally radiated to the left ear, left side of the neck, and extended to the same-side shoulder. The patient was medically healthy, with no significant medical history, no known family history of systemic diseases, and no social history of smoking, alcohol use, or other factors that could influence the current condition or treatment outcomes. Clinical and thorough extraoral examination revealed a symmetrical face and normal temporomandibular joint function. The patient demonstrated full mouth opening with a complete range of motion and symmetrical jaw movement without deviation. Clinical assessment indicated poor oral hygiene, with generalized calculus and carious lesions. Tooth number 38 exhibited signs of irreversible pulpitis, tenderness to percussion, and mild inflammation of the surrounding soft tissues. A two-point discrimination test was conducted for lip numbness, revealing incomplete sensory loss in the patient's lip. An orthopantomogram (OPG) showed a potentially concerning relationship between the inferior alveolar canal and the root of the mandibular third molar, evidenced by root darkening, canal narrowing, and upward deviation of the canal (Figure 2). Subsequent CBCT imaging confirmed entrapment of the inferior alveolar canal within the fused root of the mandibular third molar (Figure 3). Postoperatively, the extracted fragments are reconstructed using adhesive, and a simulated wire is positioned to replicate the pathway of the inferior alveolar nerve, providing additional clarity to the case (Figure 4).

**Figure 5:**
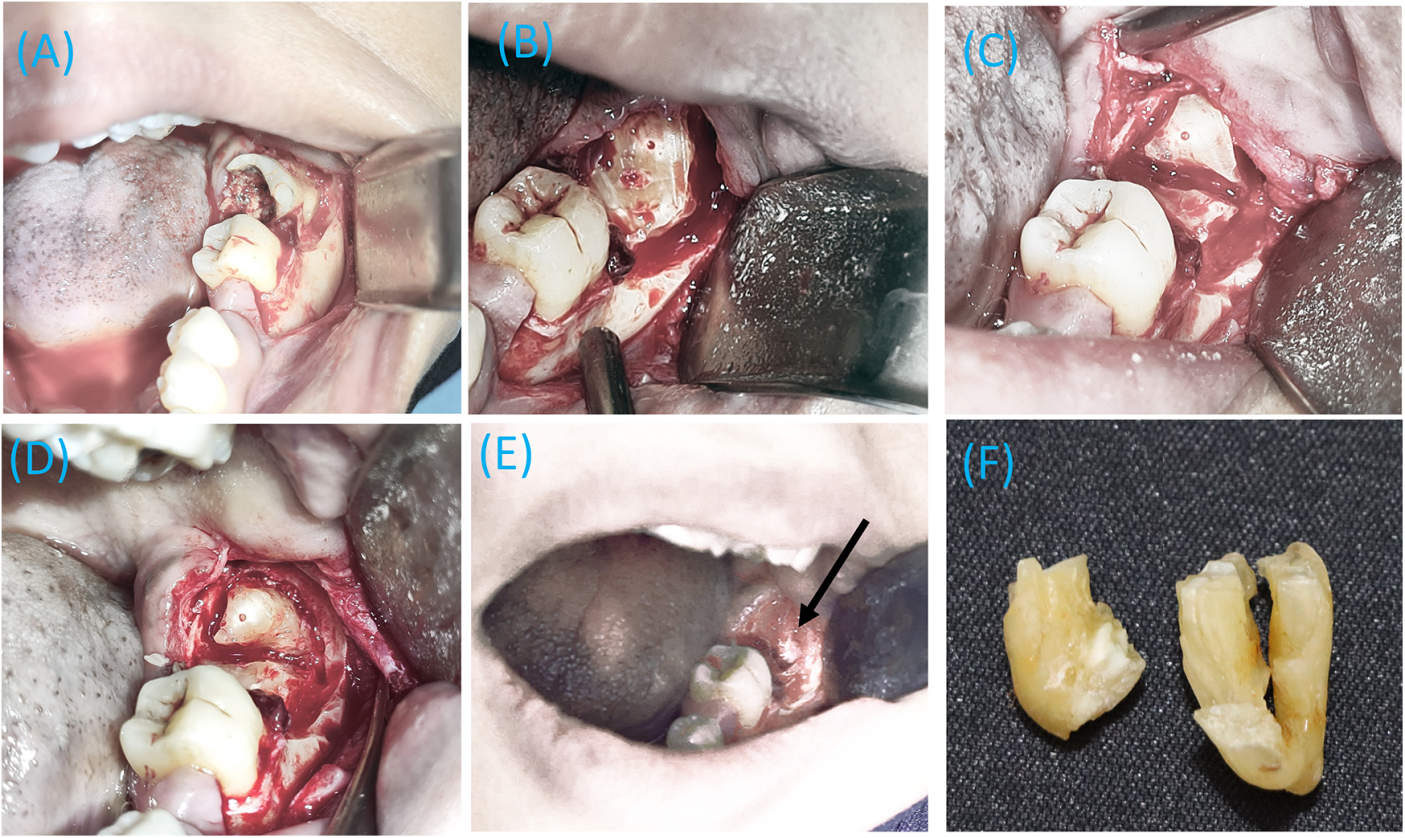
The stepwise multi-stage resection of a tooth perforated by the inferior alveolar nerve (IAN). **(A)** A modified Ward incision exposing the badly broken tooth, **(B)** Crown sectioning with buccal bone guttering, **(C)** Initial sectioning of the distal and mesial root compartments, **(D)** Final sectioning of the distal and mesial root compartments, **(E)** Complete removal of the lower left third molar, with the IAN (indicated by an arrow) visible deep in the extraction socket, and **(F)** Separated distal (short) and mesial portions of the extracted fused roots.

The surgical extraction was performed under local anesthesia. The patient was positioned in a semi-supine posture, and the area was anesthetized using two carpules of 2% lidocaine with 1:80,000 epinephrine, administered through an IAN block, along with lingual and long buccal nerve blocks. A modified Ward's incision was made using a No. 15 blade. The mucoperiosteum was reflected with a Molt periosteal elevator, and a Minnesota retractor was utilized to hold back the mucoperiosteal flaps. A stepwise, multi-stage resection technique was performed to remove the impacted tooth due to IAN entrapment (Figure 5). Specifically, buccal bone guttering was carried out, followed by crown sectioning, and a precise cut was made between the fused distal and mesial roots using a straight fissure bur. The depth of the cut, planned by CBCT, was estimated to end approximately 1 mm above the root perforation cavity to allow for controlled separation using a straight elevator. Once a crack and separation were created and confirmed between the fused roots using a wedging action with the elevator, the distal root was gently removed, followed by the mesial root. This minimally invasive approach may require identification of the fracture line if root separation is not readily perceptible.

**Figure 6:**
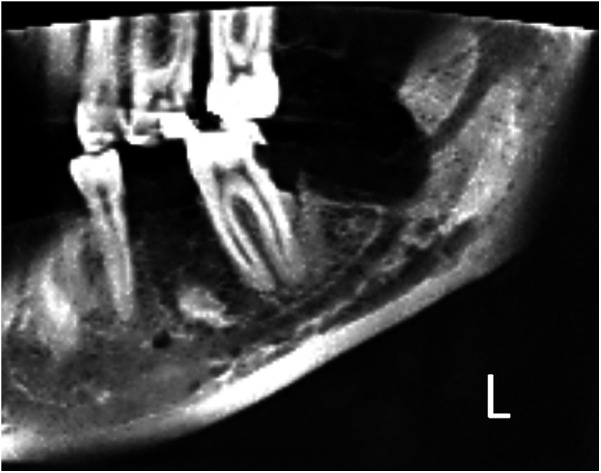
Postoperative cone beam computed tomography (part of panoramic view) shows that the left mandibular third molar was successfully extracted, with no residual roots left behind.

After the root removal, profuse bleeding from the inferior alveolar vessels occurred, which was controlled by applying physical pressure with sterile gauze. Once the bleeding was managed, the inferior alveolar bundle was identified through the socket (Figure 5). The surgical site was irrigated with saline, with continuous suction maintained throughout the procedure. The socket was then packed with a collagen sponge, and bleeding was controlled before suturing. Multiple single interrupted sutures were placed using a reverse cutting needle and a size 4-0 violet braided absorbable polyglycolic acid suture. Subsequent radiograph revealed the complete removal of the tooth with no remaining fragments (Figure 6).

Postoperative medications included Augmentin 625 mg TID for 7 days, Diclofenac potassium 50 mg TID for 7 days, Chlorohexidine mouthwash 0.12% twice daily, and Neurobion tablets, containing 100 mg of vitamin B1, 200 mg of vitamin B6, and 200 μg of vitamin B12, once daily for one month. Postoperative instructions emphasized applying cold pads within the first 24 h. The patient was advised to avoid disturbing the surgical area, refraining from contact with the tongue or fingers. A soft diet was recommended during the initial recovery phase. Postoperative oozing ceased one hour after surgery, and mild local pain at the site completely resolved by the eighth day following the procedure. At follow-up visits at two weeks, three months, and six months, the patient reported no symptoms aside for a persistent altered sensation in the left lip, which showed gradual improvement.

## 3 Discussion

This case report describes a complex pain condition in a 43-year-old female who experienced pain and persistent hypersensitivity, along with abnormal sensations and numbness radiating through the lower left lip and chin. These symptoms were caused by a combination of symptomatic pulpitis in tooth number 38 and neuropathy resulting from the entrapment of the IAN within the root of a mandibular third molar. OPG and CBCT imaging revealed nerve entrapment in the tooth substance, which indicated a carefully planned surgical extraction had to be carried out. The report is also valuable for updating current clinical knowledge and practices since there is very little available literature on such cases, and most of it is outdated. Precise preoperative evaluation of the relationship between the inferior alveolar canal and a mandibular third molar is essential for effective surgical planning. It is more accurate to refer to this condition as “IAN entrapment” rather than “IAN root perforation,” as the nerve exists before the roots of the mandibular third molar develop (10). Nonetheless, both terms have been used interchangeably in the existing literature.

Extraction of the lower third molar is a common procedure in oral and maxillofacial surgery, with a known risk of IAN damage. Identifying predictive factors for this complication is crucial for accurate surgical planning (4). Radiological evaluation is key in assessing the relationship between these structures, with panoramic imaging being a widely used method. Tantanapornkul et al. identified specific panoramic criteria, such as interruption of the mandibular canal wall or darkening of the third molar root, which are significantly correlated with direct contact observed in 3D digital images (12). Perforated third molars display a variety of radiographic appearances in OPG, with no specific signs that reliably identify these cases (6). In our case, the OPG revealed a potentially concerning relationship between the inferior alveolar canal and the root of the mandibular third molar, indicated by root darkening, canal narrowing, and an upward deviation of the canal. Notably, an upward deviation of the inferior alveolar canal has also been observed in several cases involving IAN entrapment within the roots of lower molars (6). This feature is uncommon but appears to be highly suggestive of a perforation of the root by IAN (6).

CT scans are recommended for patients whose panoramic radiographs reveal signs of a close relationship between the mandibular canal and the third molar root. It has been proposed that CBCT becomes essential when conventional radiography does not provide sufficient detail (12, 13). This was evident in our case, as the OPG results underscored the close proximity between the mandibular third molar and the canal.

In the present case, the roots were fused, with entrapment occurring in the sagittal plane between the distal and mesial segments. We adopted a stepwise, multi-stage resection technique for the removal of the involved tooth. This minimally invasive approach, which can be performed under local anesthesia, is designed to protect the IAN from injury while preserving the buccal plate. Careful handling is crucial during root separation and fragment removal. While we used a fissure bur for root separation, piezo surgery could also serve as a useful alternative, as suggested by Sato et al. (11). We left a thin, weakened apical portion intact in our technique, aiming to make the next stage of handling and removal easier while minimizing compromise to the entrapped IAN. In the next step, a gentle wedging motion with a straight elevator is applied within the prepared space between the root segments, achieving controlled separation of the remaining fused portion. This technique utilizes gentle yet steady force, and it may be beneficial to obtain a radiograph before root fragment removal to confirm a complete separation. Caution is essential when removing the separated root fragments, as their sharp edges could potentially cause trauma to the adjacent IAN.

Various surgical techniques have been proposed for managing teeth with roots enclosing the IAN. Mishra performed the tooth extraction under local anesthesia by employing a wide mucoperiosteal flap to expose the crown and removing bone from the occlusal, buccal, and distal surfaces. He visualized the neurovascular bundle through a root perforation and removed additional bone to access the tooth apex. Following this, he created a buccal window on the buccal side of the exposed root above the bundle, which allowed for gentle elevation of the nerve from the perforation and facilitated the removal of the tooth (8). Both Motamedi (9) and Sharma & Narain (7) suggested creating a buccal window, removing the crown, and then performing root sectioning and gradual elevation of the sectioned roots. Pippi proposed flap elevation followed by a pericoronal ostectomy to expose the root furcation, allowing for complete crown separation and easy mobilization of the two fragments without applying pressure to the nerve. Each root was then extracted individually, with one root ding fractured near the nerve. The apical fragment was carefully luxated and rotated around the clearly visible nerve to ensure its safe removal (10).

Coronectomy is a proposed method for managing impacted teeth closely associated with the IAN, involving the removal of only the crown while leaving the roots to gradually migrate away from the IAN (14). Orthodontic tooth extrusion is another technique that facilitates root migration before surgery (15). These methods may be less effective in cases involving the entrapment of the IAN, as the IAN migrates along with the roots (16). In fact, A migrating perforated root entrapping the IAN may lead to neurological disturbances over time due to eruption and potential nerve displacement. Moreover, removing a “coronectomized” root years later could increase the risk of injury to the entrapped nerve. Furthermore, guided bone regeneration may be proposed to prevent significant root migration in such cases (17). Alternatively, more extensive techniques like vertical ramus osteotomy and sagittal split osteotomy provide enhanced access and control, allowing better visualization of the surgical site and reducing the risk of IAN damage. However, these methods remain invasive and complex (18).

It is imperative to note that the patient initially presented with referred pain, hypersensitivity, and numbness on the lower left side of the lip prior to surgical extraction, indicating that nerve compression around the nerve trunk had already caused neuropathic pain. This was accompanied by pain due to pulpitis, highlighting the need for ongoing follow-up to monitor nerve healing, including the restoration of sensation and reduction of numbness. The entrapment of the nerve by the roots of the related tooth may have contributed to the neuropathic pain, which persisted for six months.

Pathophysiology of IAN compression and neuropathic pain involves a complex interaction between mechanical and biochemical factors that disrupt normal nerve function. The primary cause of compression neuropathy is the impairing of the microcirculation of the nerve as a result of external pressure on the nerve. The result is a failure in aerobic glycolysis, which disrupts the regulation of membrane potentials and the transmission of action potentials (19). Additionally, chronic compression can cause structural damage to the nerve, such as destruction of the endoneurium and myelin sheath, as shown in animal models (20). Furthermore, the release of inflammatory mediators caused by compression can perpetuate nerve damage and pain (21). Neuropathic pain associated with IAN compression is characterized by symptoms such as dysesthesia, paresthesia, and hypoesthesia. A difference in susceptibility to compression of nerve fibers causes these range of symptoms (22). A previous study suggested that signs of IAN compression should be considered in the differential diagnosis for unexplained facial pain, especially when impacted lower molars are located close to the nerve (7).

In fact, the presence of neuropathic pain was a key factor in our decision to remove the tooth rather than attempting other conservative treatment options. This aligns with Mishra's suggestion that an impacted tooth enclosing the IAN but not causing symptoms should not be removed (8). Unerupted and impacted teeth commonly undergo hypercementosis, and with increasing age, the bone becomes hard and sclerosed, which may even compress the nerve. Both these factors may contribute to the precipitation of painful symptoms as a result (8). Similarly, a previous case report described a 15-year-old girl presenting with a primary complaint of persistent pain in her lower molar tooth for the past four months. The pain was mild, continuous, and specifically located around the lower left second molar, radiating toward the midline of the lower lip on the same side (7).

Hypersensitivity in the lower lip or molars is a common sign of IAN injury following the surgical extraction of lower third molars (11). While most patients experience recovery from this complication, research suggests that administering intravenous steroids immediately after surgery, followed by oral steroids two days later, is widely accepted in cases of indirect nerve injuries, particularly those caused by compression of the tissues surrounding the nerve trunk within the inferior alveolar canal. This approach is favored by the anti-inflammatory and neurotropic effects of steroids (23). After follow-up visits at two weeks, three months, and six months, the patient reported no significant pain at the previous site and demonstrated a steady improvement in lip hypersensitivity and numbness.

Serious complications can occur both intra-operatively and postoperatively when extracting the mandibular third molars that enclose the inferior alveolar bundle. Hemorrhage can occur during or after surgery. Intraoperative bleeding, as observed in this case, was minimized by employing proper surgical techniques, avoiding flap tears, and reducing trauma to both the bone and surrounding soft tissue. It's crucial to control bleeding when a vessel is injured to prevent secondary hemorrhage post-surgery. The most effective method for achieving hemostasis is by applying a moist gauze pack with sufficient pressure directly to the surgical site. If the bleeding originates from the bone, the use of bone wax is recommended. Different techniques are used to help achieve local hemostasis in soft tissues such as suturing and the application of topical thrombin on a collagen sponge into the extraction socket. In the current case, the hemorrhage resulted from injury to the inferior alveolar vessels and was successfully managed by applying a sterile gauze with pressure for a few minutes, followed by the packing with collagen sponge until hemostasis was achieved.

The present case report is limited in that additional follow-up may be required to determine the outcome of the neuropathic pain. Even though the patient's symptoms, including pain in the lower left mandible, ear, and neck, were addressed through surgical extraction, slight persistent numbness remains. Given the persistent numbness, it would be beneficial to conduct additional monitoring in the long run to assess the progress of sensory recovery and manage any ongoing neuropathic pain. In conclusion, this report presents the successful extraction of a lower third molar that had been perforated by the IAN, causing neuropathic pain. A stepwise, multi-stage resection technique was employed to safely remove the affected tooth. Continuous improvement in the neuropathic pain was observed postoperatively and throughout the follow-up period.

## Data Availability

The original contributions presented in the study are included in the article/Supplementary Material, further inquiries can be directed to the corresponding author.

## References

[1] Selvi F Yildirimyan N Zuniga JR. Inferior alveolar and lingual nerve injuries: an overview of diagnosis and management. Front Oral Maxillofac Med. (2022) 4:27. 10.21037/fomm-21-8

[2] Muhsin H Brizuela M. Oral surgery, extraction of mandibular third molars. 2023. StatPearls. Treasure Island (FL): StatPearls Publishing (2024). 36508551

[3] Rood JP Shehab BA. The radiological prediction of inferior alveolar nerve injury during third molar surgery. Br J Oral Maxillofac Surg. (1990) 28(1):20–5. 10.1016/0266-4356(90)90005-6 2322523

[4] Pippi R Santoro M D'Ambrosio F. Accuracy of cone-beam computed tomography in defining spatial relationships between third molar roots and inferior alveolar nerve. Eur J Dent. (2016) 10(4):454–8. 10.4103/1305-7456.195168 28042257 PMC5166298

[5] Rud J. Third molar surgery: perforation of the inferior dental nerve through the root. Tandlaegebladet. (1983) 87(19):659–67. 6585011

[6] Chopra R Patel D Sproat C Patel V. Identifying the Polo® mint mandibular third molar: a case series. Oral Surg. (2019) 12:89–95. 10.1111/ors.12387

[7] Sharma U Narain S. Unusual facial pain secondary to inferior alveolar nerve compression caused by impacted mandibular second molar. J Indian Soc Pedod Prev Dent. (2014) 32(2):164–7. 10.4103/0970-4388 24739919

[8] Mishra YC. Entrapment of the neurovascular bundle by the roots of an impacted mandibular third molar–a case report. Br J Oral Maxillofac Surg. (1987) 25(3):261–4. 10.1016/s0266-4356(87)80028-1 3474025

[9] Motamedi MH. Impacted lower third molar and the inferior alveolar nerve. Oral Surg Oral Med Oral Pathol Oral Radiol Endod. (1999) 87(1):3–4. 10.1016/s1079-2104(99)70307-0 9927071

[10] Pippi R. A case of inferior alveolar nerve entrapment in the roots of a partially erupted mandibular third molar. J Oral Maxillofac Surg. (2010) 68(5):1170–3. 10.1016/j.joms.2009.10.007 20185220

[11] Sato Y Takamatsu K Maemura M Chao P Yamaguchi K Shirota T. Three cases of mandibular molars with roots surrounding the inferior alveolar neurovascular bundle. J Oral Maxillofac Surg Med Pathol. (2024) 36(2024):839–44. 10.1016/j.ajoms.2024.03.007

[12] Tantanapornkul W Mavin D Prapaiphittayakun J Phipatboonyarat N Julphantong W. Accuracy of panoramic radiograph in assessment of the relationship between mandibular canal and impacted third molars. Open Dent J. (2016) 10:322–32. 10.2174/187421060 27398105 PMC4920973

[13] Rodriguez Y Baena R Beltrami R Tagliabo A Rizzo S Lupi SM. Differences between panoramic and Cone Beam-CT in the surgical evaluation of lower third molars. J Clin Exp Dent. (2017) 9(2):e259–65. 10.4317/jced.53234 28210446 PMC5303328

[14] Long H Zhou Y Liao L Pyakurel U Wang Y Lai W. Coronectomy vs. total removal for third molar extraction: a systematic review. J Dent Res. (2012) 91(7):659–65. 10.1177/0022034512449346 22622663

[15] Wang Y He D Yang C Wang B Qian W. An easy way to apply orthodontic extraction for impacted lower third molar compressing to the inferior alveolar nerve. J Craniomaxillofac Surg. (2012) 40(3):234–7. 10.1016/j.jcms.2011.05.001 21641229

[16] Janovics K Soós B Tóth Á Szalma J. Is it possible to filter third molar cases with panoramic radiography in which roots surround the inferior alveolar canal? A comparison using cone-beam computed tomography. J Craniomaxillofac Surg. (2021) 49(10):971–9. 10.1016/j.jcms.2021.05.003 34090736

[17] Szalma J Soós B. Coronectomy of third molars: concerns when the roots of teeth surround the inferior alveolar neurovascular bundle. Br J Oral Maxillofac Surg. (2019) 57(10):1165–6. 10.1016/j.bjoms.2019.08.014 31477364

[18] O’Dwyer S Kelly M Pierse D. Extraction of a severely impacted mandibular third molar using a sagittal split osteotomy—a case report. J Ir Dent Assoc. (2019) 65(6):346–51. 10.58541/001c.72292

[19] Kay S McCombe D Wilks D. Pathophysiology. In: Kay S McCombe D Wilks D, editors. Oxford Textbook of Plastic and Reconstructive Surgery, Oxford Textbooks in Surgery, Online ed. Oxford: Oxford Academic (2021). 10.1093/med/9780199682874.001.0001

[20] Yoshida M. Neuropathic pain and morphologic changes in the Inferior alveolar nerve and trigeminal subnucleus caudalis in the rat. J Kyushu Dent Soc. (2003) 57(1):39. 10.2504/kds.57.39

[21] Chen SH Wu CC Lin SC Tseng WL Huang TC Yadav A Investigation of neuropathology after nerve release in chronic constriction injury of rat sciatic nerve. Int J Mol Sci. (2021) 22(9):4746. 10.3390/ijms22094746 33947104 PMC8125611

[22] Scolozzi P Lombardi T Jaques B. Successful inferior alveolar nerve decompression for dysesthesia following endodontic treatment: report of 4 cases treated by mandibular sagittal osteotomy. Oral Surg Oral Med Oral Pathol Oral Radiol Endod. (2004) 97(5):625–31. 10.1016/S1079210404000502 15153877

[23] On SW Cho SW Byun SH Yang BE. Clinical significance of intraoperative exposure of inferior alveolar nerve during surgical extraction of the mandibular third molar in nerve injury. J Clin Med. (2021) 10(19):4379. 10.3390/jcm10194379 34640397 PMC8509309

